# Semantic–Physical Sensor Fusion for Safe Physical Human–Robot Interaction in Dual-Arm Rehabilitation

**DOI:** 10.3390/s26051510

**Published:** 2026-02-27

**Authors:** Disha Zhu, Xuefeng Wang, Shaomei Shang

**Affiliations:** 1 School of Nursing, Peking University, Beijing 100871, China; 1901213574@pku.edu.cn; 2 School of Advanced Manufacturing and Robotics, Peking University, Beijing 100871, China; wang_xf@pku.edu.cn

**Keywords:** rehabilitation robotics, physical human–robot interaction, large language models, multimodal fusion, state estimation

## Abstract

A safe physical human–robot interaction (pHRI) in rehabilitation requires reliable perception and low-latency decision making under heterogeneous and unreliable sensor inputs. This paper presents a multimodal sensor-fusion-based safety framework that integrates physical state estimation, semantic information fusion, and an edge-deployed large language model (LLM) for real-time pHRI safety control. A dynamics-based virtual sensing method is introduced to estimate internal joint torques from external force–torque measurements, achieving a normalized mean absolute error of 18.5% in real-world experiments. An asynchronous semantic state pool with a time-to-live mechanism is designed to fuse visual, force, posture, and human semantic cues while maintaining robustness to sensor delays and dropouts. Based on structured multimodal tokens, an instruction-tuned edge LLM outputs discrete safety decisions that are further mapped to continuous compliant control parameters. The framework is trained using a hybrid dataset consisting of limited real-world samples and LLM-augmented synthetic data, and evaluated on unseen real and mixed-condition scenarios. Experimental results show reliable detection of safety-critical events with a low emergency misdetection rate, while maintaining an end-to-end decision latency of approximately 223 ms on edge hardware. Real-world experiments on a rehabilitation robot demonstrate effective responses to impacts, user instability, and visual occlusions, indicating the practical applicability of the proposed approach for real-time pHRI safety monitoring.

## 1. Introduction

Multi-modal sensory perception is critical for intelligent robotics. This includes vision, force, and physiological monitoring. Industrial robots operate in structured environments, but service robots must interpret complex human behaviors and safety constraints during physical Human–Robot Interaction (pHRI) [1,2]. This requirement is critical in physical rehabilitation. Here, the robot acts as a cognitive partner rather than a simple mechanical actuator [3,4,5,6]. It must understand the user’s physiological state and intent [7,8,9,10,11].

However, current robotic implementations lag behind these requirements [12,13]. Rehabilitation robots offer high precision and adaptive feedback [14,15,16,17]. Although mechanical solutions like Series Elastic Actuators have successfully addressed physical compliance to some extent [18,19,20,21,22], it is still hard for robots to have cognitive adaptability in unstructured home environments. Raw data from a single sensor cannot provide robust state estimation [23,24,25]. To address this limitation, various multi-sensor fusion techniques have been investigated. Traditional approaches utilize probabilistic methods, such as Kalman Filters, to merge kinematic and dynamic data [26,27]. More recent studies employ deep neural networks to fuse visual and haptic modalities [28,29,30]. However, these methods typically focus on low-level feature integration and lack the high-level reasoning capabilities required to interpret complex user behaviors in unstructured scenarios. This creates bottlenecks at two levels: physical perception and semantic understanding.

At the physical perception level, external haptic feedback does not accurately reflect the internal human physiological state. The mapping from sensor readings to joint torques is non-linear [31]. In dynamic rehabilitation, the interaction force combines human joint effort with system dynamics [32,33,34]. For example, different limb configurations result in different internal torques even if the external contact force is identical. High force readings may result from gravity rather than active resistance. Direct reliance on raw force thresholds causes misinterpretations. Therefore, we need a model-based physical observer (inverse dynamics) to decouple disturbances and recover the objective intrinsic state [35,36].

At the semantic level, kinematic modalities like vision suffer from ambiguity without physical constraints [2,37]. Visual algorithms capture skeletal poses but struggle to distinguish geometrically similar scenarios. For instance, “reclining on a chair” and “falling onto the floor” look similar. Both show a horizontal trunk and lowered center of mass [38]. Without cross-modal verification from other sensors, the vision system itself cannot correctly distinguish the scenario [39,40,41].

As the rehabilitation ecosystem expands, the integration of various sensors, such as wearable devices and environmental IoT devices, leads to exponential growth in semantic information combinations. Traditional finite state machines encounter the problem of “combinatorial explosion” when facing multimodal combinations, requiring the writing of thousands of if-else rules, and appear particularly fragile when dealing with undefined edge cases [42,43]. To address this issue, the control framework needs to introduce Large Language Models (LLMs) as the reasoning core [44,45]. Using the LLM’s semantic space compression and zero-shot generalization capabilities, LLMs can interpret heterogeneous sensor data and generate adaptive control strategies for complex unseen scenarios, handling all unknown logical combinations with a single network [46].

To solve this problem, we propose a multi-level sensor fusion dual-arm rehabilitation robot framework. This framework unifies the “Physical Fusion” of vision and haptics with the “Semantic Fusion” of Large Language Models [47]. The “Physical Fusion” establishes a method for observing human internal torque based on multi-sensor perception. The “Semantic Fusion” drives the adaptive adjustment of control hyperparameters [48,49,50].

We constructed a system with two 6-DOF robotic arms, depth cameras, and force sensors. Through inverse dynamics, the system calculates the human joint internal torque. Our experiments show an average estimation error of 18.5% for this physiological parameter. For the cognitive core, we fine-tuned a Qwen3-1.7B model via LoRA. This enables the robot to make comprehensive judgments based on multi-source information in rehabilitation scenarios and output structured control commands. Experiments demonstrate that the fine-tuned model achieves an accuracy of 98.5% on the test set with an inference speed of about 4.48 Hz.

In the proposed framework, these two fusion layers are not independent. The physical fusion layer acts as a **Feature Extractor**. Raw sensor data contains noise from gravity and robot inertia, which leads to poor LLM generalization. By resolving the normalized human internal torque first, we provide the LLM with configuration-independent physical semantics. This allows the LLM to focus on logical reasoning (e.g., distinguishing between a spasm and a fall), thereby achieving robust generalization capabilities even in unseen scenarios.

It is worth noting that while we validate our framework using a dual-arm robot for knee rehabilitation, the proposed method is not limited to this specific configuration. The choice of a redundant dual-arm system is strategic; it addresses the composite rehabilitation needs prevalent in the elderly population, where patients often require therapy across multiple joints rather than a single site [51,52]. Instead of deploying multiple specialized machines, this system serves as a proof-of-concept for **“software-defined rehabilitation”**, enabling a single general-purpose robot to adapt to various anatomical targets and therapeutic protocols through software reconfiguration. Furthermore, the knee rehabilitation task represents a high-torque, dynamically complex scenario, serving as a rigorous benchmark to demonstrate the robustness of our semantic–physical fusion framework, which can be readily extended to other robotic systems and rehabilitation tasks.

The remainder of this paper is organized as follows. Section 2 details the system architecture. Section 3 describes the physical modeling fusion method. Section 4 introduces the LLM-based semantic fusion. Section 5 presents the pHRI control and validation. Section 6 concludes the paper.

## 2. System Architecture and Implementation

**Figure 1:**
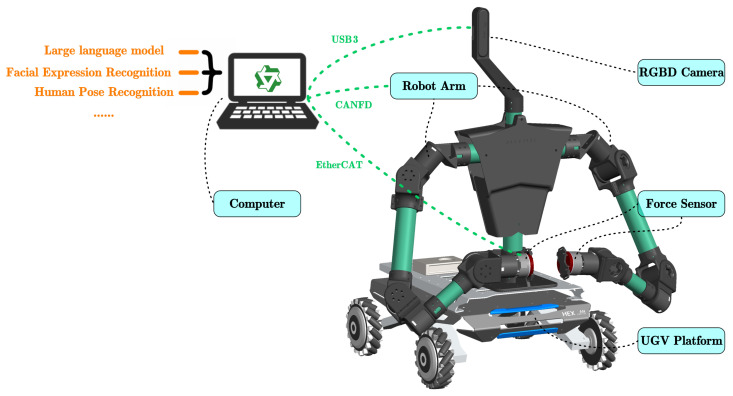
Key components of the rehabilitation robot system.

The system has two main parts: a rehabilitation robot and a prosthetic verification platform. As shown in Figure 1, the rehabilitation robot contains three modules: mobility, actuation, and perception-computation.

The mobility and actuation modules form the robot’s body. We built the mobile base on the HEXMAN-MARK1 platform. It uses Mecanum wheels with integrated odometry and Inertial Measurement Units (IMU) for movement. The dual-arm system uses two 6-Degree-of-Freedom (6-DoF) robotic arms with a spherical-wrist configuration. High-torque density brushless motors (DM4340P and DM4310, Maxon Motor, Tagerwilen, Switzerland) drive the joints. These motors support Quasi-Direct Drive (QDD) control to ensure whole-body compliance. For command execution, we used an STM32-based data acquisition card (STMicroelectronics, Geneva, Switzerland). It communicates via an independent CAN FD bus (Bosch; Gerlingen, Germany) at 250 Hz to maintain stable torque loop control.

The multi-modal perception and computing system includes the following components:

1. **Visual System:** An Orbbec 336L RGB-D depth camera (Orbbec, Shenzhen, China) is mounted on the robot’s head to capture environmental context. It streams video at a resolution of 1920×1080 (30 fps), with depth data synchronized via USB to the host for real-time skeletal tracking using YOLO-POSE.
2. **Haptic System:** Two XJC-XD60EC 6-DoF force/torque sensors (XJC Electronics, Shenzhen, China) are installed at the end-effectors to capture interaction forces. Crucially, these sensors communicate via an EtherCAT bus (EtherCAT Technology Group, Nuremberg, Germany) with a dedicated master station (ZMC60E), ensuring strictly deterministic data acquisition at 1 kHz with minimal jitter.
3. **Computing Core:** The central processing unit is a mobile workstation equipped with an **NVIDIA GeForce RTX 5060 Laptop GPU**. This platform acts as the sensor fusion hub, aggregating data from heterogeneous interfaces, including USB (vision), UDP (force sensors via EtherCAT bridge), CAN (mobile base), and CAN FD (robotic arms). The system architecture adopts a hybrid computing strategy; the CPU handles high-frequency dynamics and control tasks, while the GPU provides sufficient CUDA cores to support the real-time inference of the fine-tuned Large Language Model (LLM) alongside other lightweight networks such as YOLO-POSE.

**Figure 2:**
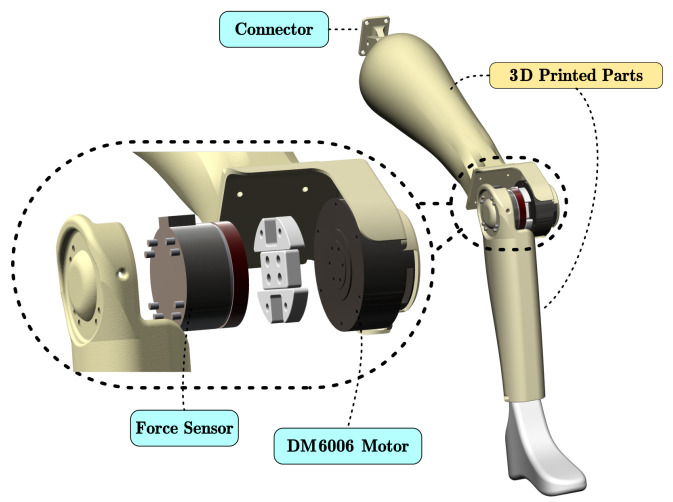
Key components of the robotic prosthetic verification platform.

We developed a robotic prosthetic verification setup to test the algorithms (Figure 2). A QDD brushless servo motor (DM6006, CAN bus at 100 Hz) actuates the knee joint of the prosthetic limb to simulate human joint impedance. We 3D-printed the limb structure using resin and mounted it on a fixed frame. We placed a force sensor (identical to the robot’s sensor) in the knee joint. This sensor measures the ground truth internal torque $\tau_{\mathrm{limb}\text{-}\mathrm{internal}\text{-}\mathrm{real}}$.

**Figure 3:**
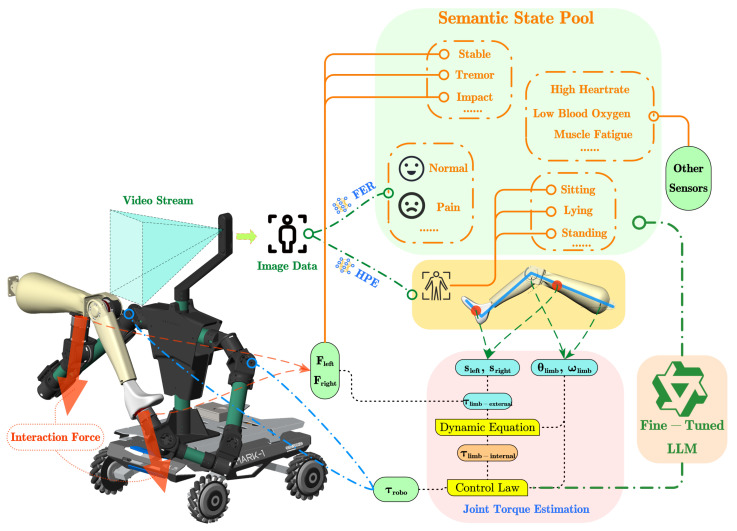
Operational diagram and data flow of the integrated system.

Figure 3 illustrates the hardware integration and data flow framework, where visual, haptic, and auxiliary signals are synchronized and fused. A dual-path processing strategy is adopted; the semantic path updates a state pool for LLM reasoning, while the physical path combines kinematic and dynamic data to estimate human joint torques. In the physical formulation, the interaction forces measured by the robot’s end-effectors are denoted by $F_{\mathrm{left}}$ and $F_{\mathrm{right}}$, and the robotic joint torques by $\tau_{\mathrm{robo}}$. The limb state is characterized by joint angles $\theta_{\mathrm{limb}}$ and angular velocities $\omega_{\mathrm{limb}}$ relative to the support points s. Based on these parameters, the system distinguishes the external torque applied by the robot, $\tau_{\mathrm{limb}\text{-}\mathrm{external}}$, from the user’s physiological internal torque, $\tau_{\mathrm{limb}\text{-}\mathrm{internal}}$.

We split the data flow based on signal type. Facial Expression Recognition (FER) extracts emotional states (e.g., ‘Pain’, ‘Normal’) for the semantic pool. Human Pose Estimation (HPE) has two roles. It sends geometric features to the inverse dynamics model, and it registers high-level pose descriptions (e.g., ‘Sitting’, ‘Lying’) in the semantic pool. Similarly, force sensor signals drive the dynamics observer for low-level control. At the same time, interpreted interaction events (e.g., ‘Stable’, ‘Impact’) provide context for the semantic decision layer.

## 3. Physical Fusion Layer: Dynamics-Based Torque Observation

The primary goal of the physical sensing module is to estimate the user’s internal joint torque. This metric reflects the true physiological state. In dynamic rehabilitation, raw force sensor readings at the end-effector contain noise from mechanical dynamics, such as inertial forces and gravitational components. These factors distort the measurement of active human effort. To remove these disturbances, we applied a **“Physical Fusion”** strategy. We modeled the human limb (or the verification platform) as a non-uniform two-link system. We determined the mass distribution parameters through preliminary experiments.

### 3.1. Dynamics of the Coupled System

We model the human limb (or the verification prosthetic platform) as a two-link system to separate physiological intent from mechanical measurements. We parameterized the geometric configuration and mass distribution for the dynamic analysis.

**Figure 4:**
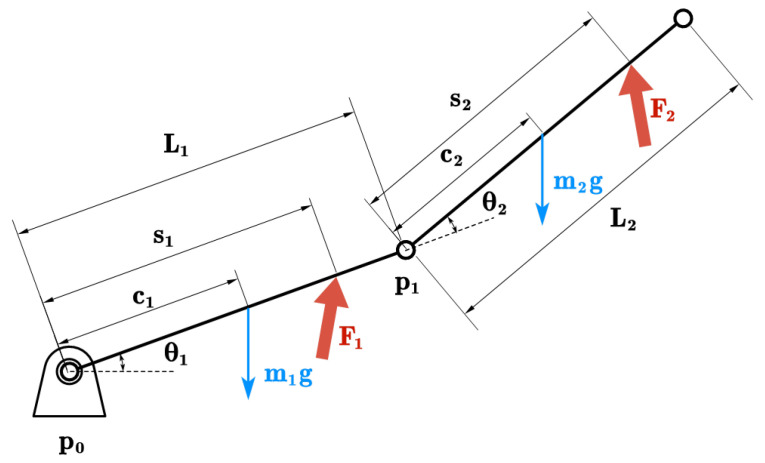
Kinematic model and vector parameterization of the two-link limb system.

Figure 4 shows the system’s spatial definitions. $p_{i}$, $c_{i}$, $m_{i}$, and $s_{i}$ denote the position vectors of the link endpoints (joints), centers of mass, the masses of the links, and sensor interaction points, respectively. Additionally, $F_{i}$ denotes the interaction forces acting on the limb segments.

We define the generalized coordinates as the joint configuration vector $q_{\mathrm{limb}}={\left[\theta_{1},\theta_{2}\right]}^{⊤}$, which corresponds to the hip and knee angles. Using the Euler–Lagrange equation, we express the dynamic model as:

(1) $$M_{\mathrm{limb}}\left(q_{\mathrm{limb}}\right){\ddot{q}}_{\mathrm{limb}}+C_{\mathrm{limb}}\left(q_{\mathrm{limb}},{\dot{q}}_{\mathrm{limb}}\right){\dot{q}}_{\mathrm{limb}}+G_{\mathrm{limb}}\left(q_{\mathrm{limb}}\right)=\tau_{\mathrm{limb}\text{-}\mathrm{external}}+\tau_{\mathrm{limb}\text{-}\mathrm{internal}}$$

$M_{\mathrm{limb}}$ is the inertia matrix, $C_{\mathrm{limb}}$ represents Coriolis and centrifugal effects, and $G_{\mathrm{limb}}$ is the gravity vector. The right-hand side decomposes the total torque into external mechanical interaction ($\tau_{\mathrm{limb}\text{-}\mathrm{external}}$) and the user’s internal physiological torque ($\tau_{\mathrm{limb}\text{-}\mathrm{internal}}$).

We identified the dynamic parameters ($m_{1},m_{2},\ldots$) offline. This allows us to calculate the inertial and gravitational components in real time. We observe the internal torque by subtracting the passive dynamics and external loads from the total system dynamics.

### 3.2. Synthesizing the Internal Torque Observer

We use an explicit geometric formulation based on the kinematic chain in Figure 4. By analyzing the force vectors and lever arms relative to the joint frames, the generalized external torque $\tau_{\mathrm{limb}\text{-}\mathrm{external}}\in R^{2}$ is derived as the vector of scalar moments acting along the joint axes (*z*-axis):

(2) $$\tau_{\mathrm{limb}\text{-}\mathrm{external}}=\left[\begin{array}{c} {\left(s_{1}\times F_{1}+\left(p_{1}+s_{2}\right)\times F_{2}\right)}_{z}\\ {\left(s_{2}\times F_{2}\right)}_{z} \end{array}\right]$$

where ${\left(\cdot \right)}_{z}$ denotes the scalar projection onto the joint rotation axis. This formulation ensures that the dimensions of the external torque vector align with the generalized coordinates $q_{\mathrm{limb}}$.

After reconstructing the load terms, we apply the inverse dynamics model to the observer. By rearranging the dynamic equilibrium equation, we solve for the user’s internal torque:

(3) $$\tau_{\mathrm{limb}\text{-}\mathrm{internal}}=M_{\mathrm{limb}}\left(q_{\mathrm{limb}}\right){\ddot{q}}_{\mathrm{limb}}+C_{\mathrm{limb}}\left(q_{\mathrm{limb}},{\dot{q}}_{\mathrm{limb}}\right){\dot{q}}_{\mathrm{limb}}+G_{\mathrm{limb}}\left(q_{\mathrm{limb}}\right)-\tau_{\mathrm{limb}\text{-}\mathrm{external}}$$

This calculated variable $\tau_{\mathrm{limb}\text{-}\mathrm{internal}}$ acts as the “Physical Token”. It represents the normalized limb torque decoupled from the robot’s mechanical influence.

#### Quantitative Accuracy Analysis

**Figure 5:**
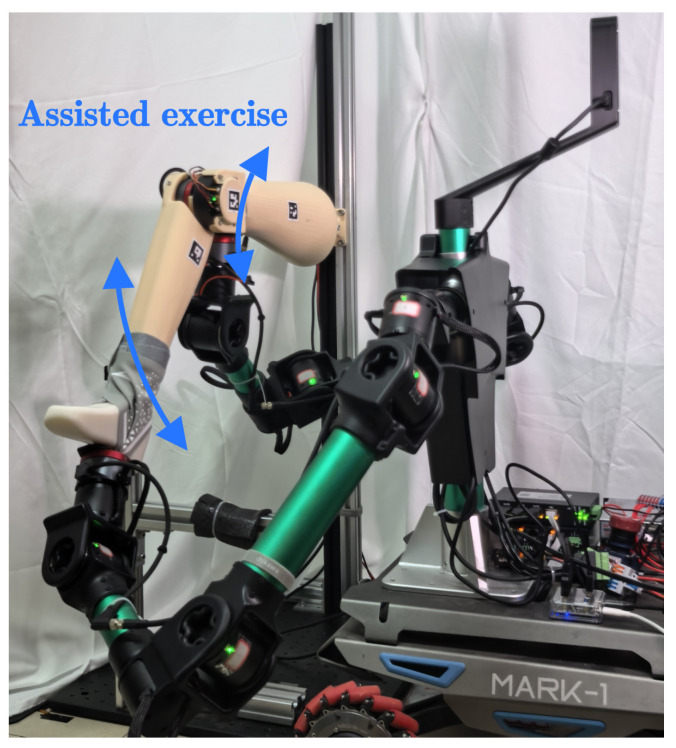
Experimental scenario: a dual-arm robot manipulating the instrumented prosthesis to validate internal torque estimation.

To validate the algorithm, we built a physical verification setup. A dual-arm robot manipulates an instrumented prosthetic leg to simulate passive rehabilitation trajectories, as shown in Figure 5.

We use the Normalized Mean Absolute Error (NMAE) as the primary metric. It is the mean absolute deviation normalized by the peak-to-peak (PP) amplitude of the ground truth signal:

(4) $$\mathrm{NMAE}=\frac{1}{n\cdot {\mathrm{PP}}_{\tau }}\sum_{i=1}^{n}\left|\tau_{\mathrm{ground}\_\mathrm{truth},i}-{\hat{\tau }}_{\mathrm{observed},i}\right|$$

where $\tau_{\mathrm{ground}\_\mathrm{truth},i,i}$ is the ground truth torque measured by the prosthesis’s internal sensors, and ${\hat{\tau }}_{\mathrm{observed},i}$ is the observer’s estimate. *n* is the total number of sampling points, and ${\mathrm{PP}}_{\tau }$ denotes the peak-to-peak amplitude of the ground truth torque signal.

**Figure 6:**
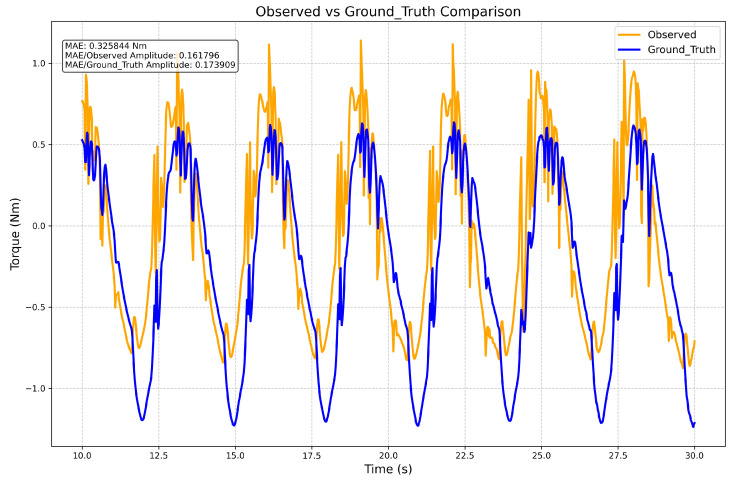
Estimation accuracy: comparison between the observed limb torque (orange) and the ground truth sensor data (blue).

Figure 6 shows the comparison results. The observer outputs (orange) track the ground truth data (blue) despite structural uncertainties.

Figure 6 presents the results for a typical rehabilitation trajectory with a period T=3s and an amplitude A=0.2rad. We conducted 24 experiments with different periods (T = 3 s, 4 s, 5 s, 6 s) and amplitudes (A = 0.1, 0.2, 0.3). The observer achieved a mean absolute error (MAE) of 0.336 Nm and an average NMAE of 18.5% for robotic prosthesis torque estimation. This discrepancy comes from four main physical factors:

1. **Unmodeled Actuator Friction:** The simplified dynamic model does not fully capture non-linear actuator characteristics, such as dry friction and stiction in the motor and gearbox.
2. **Kinematic Calibration Residuals:** The kinematic parameters of the dual-arm robot have finite calibration accuracy. This introduces errors in the geometric Jacobian and gravity compensation terms.
3. **Contact Instability:** Non-rigid contact and micro-slippage at the robot–prosthesis contact point cause transient deviations in the estimated force application point. This leads to errors in the lever arm calculation.
4. **Localization and Calibration Uncertainties:** Leg tracking uses ArUco markers. Precise alignment is affected by cumulative errors: noise in visual pose estimation (approx. 0.036 rad MAE), calibration inaccuracies in the robot base frame, and geometric differences between the marker’s theoretical position and its actual placement.

The dual-arm robot used in this study is a laboratory-developed prototype. While its kinematic calibration exhibits finite residuals compared to high-precision industrial manipulators, the system’s consistent performance across multiple trials demonstrates that the method is not fragile to these hardware-level imperfections. For human subjects, inertial parameters were estimated using standard anthropometric scaling laws (similar to OpenSim), in contrast to the experimental identification used for the verification platform. Given that rehabilitation exercises typically involve low angular velocities and accelerations, the dynamic behavior described in (3) is dominated by the gravitational vector $G_{\mathrm{limb}}$ rather than the higher-order inertial or Coriolis terms. Since $G_{\mathrm{limb}}$ depends linearly on the mass parameters, any estimation errors in the anthropometric data result in bounded, linear offsets in $\tau_{\mathrm{limb}\text{-}\mathrm{internal}}$, preventing the divergent instability often seen in high-speed control.

In terms of accuracy, most existing work validates torque estimation using OpenSim simulations [53], which inherently bypasses real-world sensor noise and transmission non-linearities. While physical sensing approaches like wearable ultrasound can achieve approximately 7.6% error under ideal conditions [54], achieving 18.5% NMAE on a full dynamic robotic platform represents a robust baseline for real-world interaction. Human motor control exhibits high variability, and the system’s goal is reliable semantic classification (e.g., distinguishing spasms from resistance) rather than surgical force tracking. Since the torque disparity between normal motions and hazardous anomalies is substantial, the current accuracy falls well within physiological tolerance for safety-critical decision making. These results confirm that the model-based observer effectively decouples passive mechanical effects (e.g., limb gravity) from active joint torque to recover the user’s true internal state, with potential for further accuracy gains through industrial-grade calibration.

## 4. Semantic-Physical Fusion and Intelligent Decision

This section describes the system’s intelligence layer. We propose an asynchronous fusion framework that separates sensor sampling rates from decision-making frequencies. This allows a Fine-Tuned Large Language Model to generate robust control decisions on edge hardware.

### 4.1. Asynchronous Semantic State Pool

**Figure 7:**
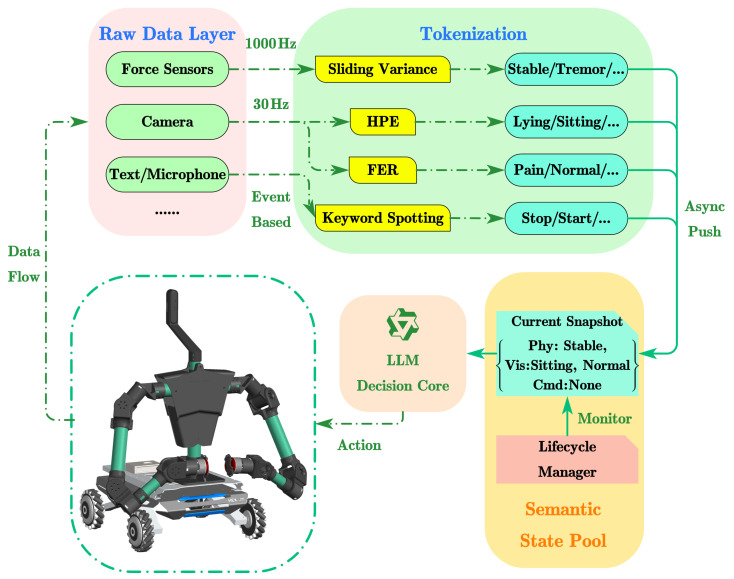
Data flow architecture of the asynchronous semantic state pool. Heterogeneous sensor data (visual, physical, user input) are independently processed into discrete semantic tokens and aggregated into a unified state snapshot for the LLM.

In rehabilitation scenarios, sensors operate at different frequencies. Force sensors run at 1 kHz, visual pose estimation at 30 Hz, motors at 250 Hz, and other monitors update sparsely. To handle this, we designed a **Dynamic Semantic State Pool** on the central computer (Figure 7).

The State Pool stores the latest semantic status of all subsystems as key–value pairs. The process includes:

1. **Signal Discretization and Tokenization:** Raw sensor data are continuous and high-frequency, which is unsuitable for direct LLM ingestion. We employ a sliding-window statistical method to convert these signals into semantic tokens:
  - **Physical Tokens (from Section 3):** The internal torque $\tau_{\mathrm{limb}\text{-}\mathrm{internal}}$ derived in Equation (3) is processed to quantify interaction stability. We calculate the sliding variance $\sigma_{\tau_{\mathrm{error}}}^{2}$ over a window W=50 ms:
    (5) $$\sigma_{\tau_{\mathrm{error}}}^{2}\left(t\right)=\frac{1}{W}\sum_{k=t-W}^{t}{\left(\tau_{\mathrm{internal}}\left(k\right)-\bar{\tau }\right)}^{2}$$
    where W=50ms is the window size, and $\bar{\tau }$ represents the moving average of the internal torque within this window. The variance is mapped to discrete tokens based on thresholds $\left\{\delta_{1},\delta_{2}\right\}$ calibrated on a 30-trial pilot dataset:
    (6) $${\mathrm{Token}}_{\mathrm{phy}}=\left\{\begin{array}{cc} \mathrm{STABLE}, & \mathrm{if}\;\sigma_{\tau_{\mathrm{error}}}^{2}\leq \delta_{1}\\ \mathrm{TREMOR}, & \mathrm{if}\;\delta_{1}<\sigma_{\tau_{\mathrm{error}}}^{2}\leq \delta_{2}\\ \mathrm{IMPACT}, & \mathrm{if}\;\sigma_{\tau_{\mathrm{error}}}^{2}>\delta_{2} \end{array}\right.$$
    Specifically, $\delta_{1}$ is set to $\mu_{\mathrm{noise}}+3\sigma_{\mathrm{noise}}$ (3-sigma rule) to filter baseline noise, where $\mu_{\mathrm{noise}}$ and $\sigma_{\mathrm{noise}}$ are the mean and standard deviation of the sensor noise measured during static calibration. $\delta_{2}$ is selected to maximize the margin between oscillatory tremors and high-energy impact spikes.
  - **Human Pose Tokens:** Keypoint coordinates from YOLO-POSE are analyzed for geometric anomalies. For instance, when the vertical difference between the hip and head keypoints falls below a calibrated threshold indicative of a supine posture, the token Visual: USER_LYING_DOWN is pushed to the pool.
  - **Facial Expression Tokens:** The vision system runs a lightweight FER module to classify user emotions. When the confidence score for a critical state (e.g., ‘Pain’ or ‘Fatigue’) exceeds a threshold of 0.8, a corresponding semantic token (e.g., Face: PAIN) is generated and updated in the state pool.
  - **Instruction Tokens:** User commands (via voice or text interface) are parsed into intent tokens. For example, the command “Stop moving” immediately triggers a high-priority Cmd: STOP token, overriding other behaviors.
2. **Asynchronous Update:** Each sensor module pushes updates to the pool independently. The LLM acts as a consumer, querying the current snapshot of the pool for inference.
3. **Lifecycle Management:** To prevent stale data from influencing decisions (e.g., if the camera is occluded), a time-to-live (TTL) mechanism is implemented. Any state token not updated within **5.0 s** is automatically invalidated and removed from the pool.

This mechanism merges high-frequency physical data with low-frequency semantic context without blocking.

### 4.2. Edge-Native Instruction-Tuned Model

Real-time robotic control requires structured outputs and deterministic latency. General cloud LLMs often fail these requirements due to network variability. Therefore, we deployed the **Qwen3-1.7B Large Language Model** on the local hardware (NVIDIA RTX 5060 Laptop GPU).

We used **Instruction Fine-Tuning** instead of prompt engineering. This provides two advantages:

1. **Structured Output Reliability:** General LLMs typically produce verbose “chain-of-thought” narratives. Through fine-tuning, our model is constrained to output *only* the control action codes (“0”, “1”, or “2”), eliminating the need for complex post-processing regex parsing and ensuring machine-readable stability.
2. **Low-Latency Inference:** By optimizing the model scale (1.7B parameters) and executing locally, we achieved an average inference latency of approximately 223 ms on the laptop. Including sensor acquisition and bus communication, the total control loop latency is controlled within 240 ms. Since rehabilitation scenarios primarily involve physical human–robot interaction frequencies below 1 Hz (period >1 s), this response speed falls well within the safety margins for smooth control.

The fine-tuned model serves as a function with semantic understanding capabilities and generalization ability, mapping the chaotic multimodal state space of the rehabilitation environment to a discrete, safe action space with structured outputs.

### 4.3. Data Augmentation and Training Pipeline

High-quality, domain-specific data is scarce in robotic rehabilitation. To solve this, we used a **“Seed-Augment-Train”** strategy. We used a foundation model to synthesize a robust dataset.

#### 4.3.1. Dataset Construction

The process involved three stages:

1. **Expert Seed Creation:** We manually curated a small set of approximately 20 “Instruction-Input-Output” seed samples covering typical rehabilitation scenarios (e.g., normal training, fatigue indications, sudden impacts, falls).
2. **LLM-Driven Augmentation:** Using a large-scale foundation model (QWEN3-MAX), we generated 2000 diverse training samples based on the seed patterns. The prompt constrained the generator to introduce variations in semantic phrasing (e.g., rephrasing “Seat: not detected” to “Seat: user stood up”) while maintaining strict logical consistency with the safety protocols.
3. **Standardization:** The augmented data was formatted into a strict JSON structure for Supervised Fine-Tuning (SFT).

**Figure 8:**
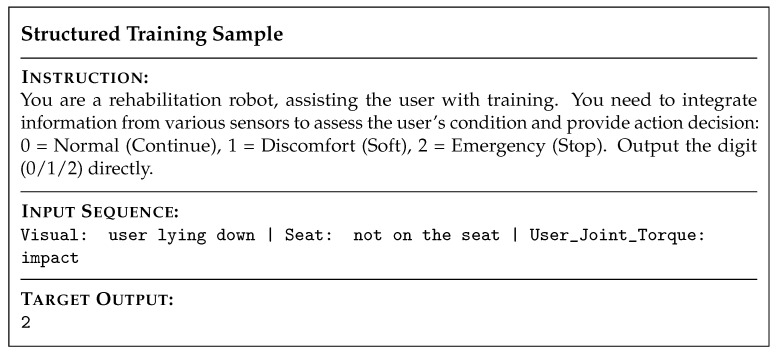
Visualization of the prompt structure. The input aggregates multimodal semantic tokens, while the instruction enforces a standardized action space.

We standardized the data format to maintain consistency between training and inference. Figure 8 shows a representative sample.

#### 4.3.2. Fine-Tuning Implementation

We fine-tuned Qwen3-1.7B on the synthetic dataset using Low-Rank Adaptation (LoRA). We applied LoRA modules to the linear layers in both the attention mechanisms and feed-forward networks. Table 1 details the hyperparameter configuration.

We selected a scaling ratio of α/r=4. This configuration strengthens the weight updates for the rehabilitation data, helping the model learn the semantic–action mapping while avoiding the instability of full-parameter fine-tuning.

#### 4.3.3. Quantitative Evaluation

We evaluated the fine-tuned model on a held-out test set of 2000 samples. The model achieved an accuracy of 98.5% (1970/2000).

**Inference Speed:**

The average inference latency on the local edge GPU was **0.223 s**. Beyond average performance, tail latency is critical for safety guarantees to prevent sporadic delays. The system achieved a **P95 latency of [0.227] s** and a **P99 latency of [0.255] s**. This distribution confirms that even in outlier cases, the control loop maintains a reaction speed within the acceptable safety margin. In the context of physical human–robot interaction (pHRI), this latency is comparable to the human physiological haptic response time, which typically ranges from 198 ms to 313 ms [55]. Furthermore, since the LLM-based cognitive core is utilized for high-level control strategy switching (e.g., transition from active to passive mode) rather than low-level real-time trajectory servoing, this delay remains transparent to the user and does not compromise the stability of the interaction.

**Error Analysis for Safety (Action 2):**

We analyzed the confusion matrix directly to assess safety risks:

- **False Positives:** Out of 1121 samples predicted as “Stop”, 4 were actually “Soft” cases.
- **False Negatives:** Out of 1129 actual “Stop” cases, 12 were misclassified. All 12 were labeled as “Soft” (Action 1), and none were labeled as “Continue” (Action 0).

**Figure 9:**
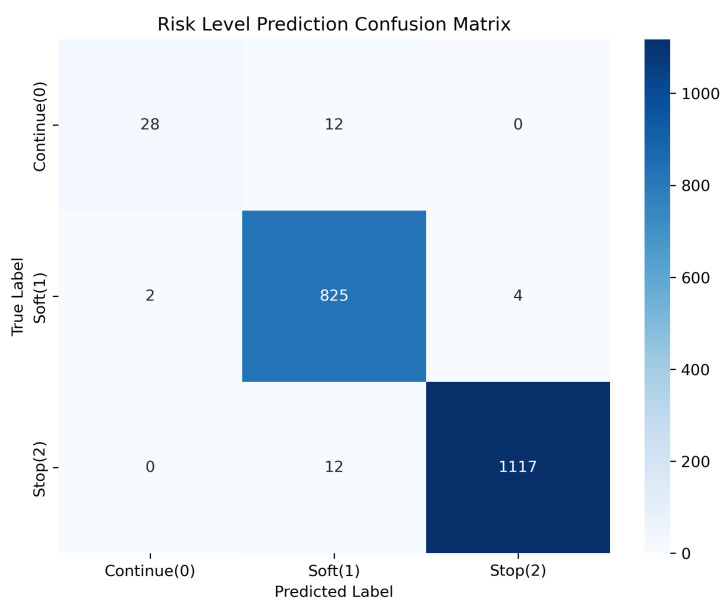
Confusion matrix on the held-out test set (N = 2000).

The data (Figure 9 and Table 2) indicates that while the model is not perfectly accurate, the errors in this test set were confined to adjacent safety levels.

#### 4.3.4. Supplementary Experiments on Small-Sample Data

We performed two additional checks using limited datasets to observe model behavior on non-synthetic data.

1. Expert Seed Data (N = 20)

**Figure 10:**
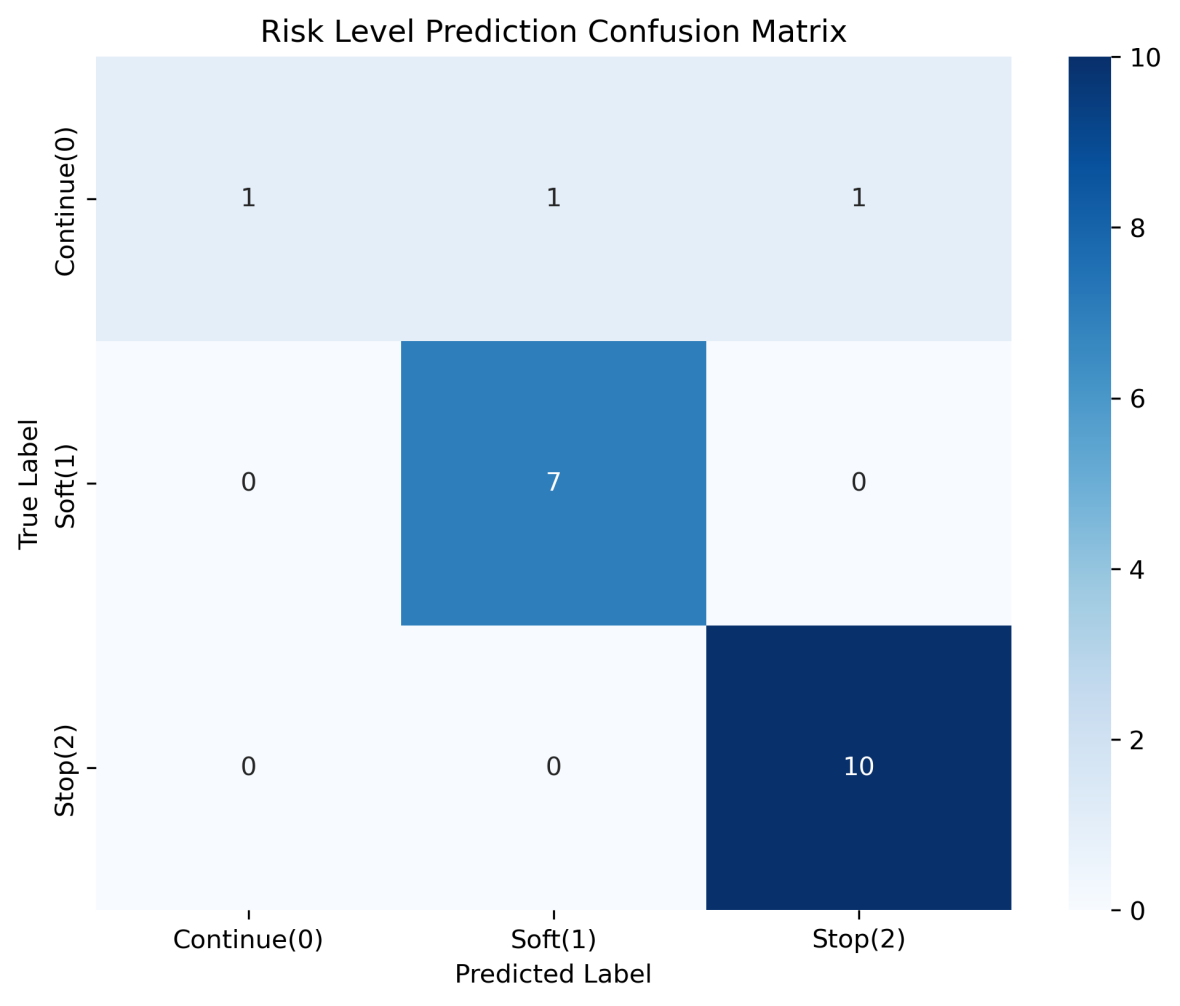
Performance on manually curated seed data (N = 20).

We tested the model on the 20 initial manual seed samples as shown in Figure 10. The accuracy was 90.0% (18/20).

- The 2 errors were: one “Continue” classified as “Soft”, and one “Continue” classified as “Stop”.
- In this specific set, all 10 actual “Stop” samples were correctly classified.

2. Unseen Vocabulary (N = 10)

**Figure 11:**
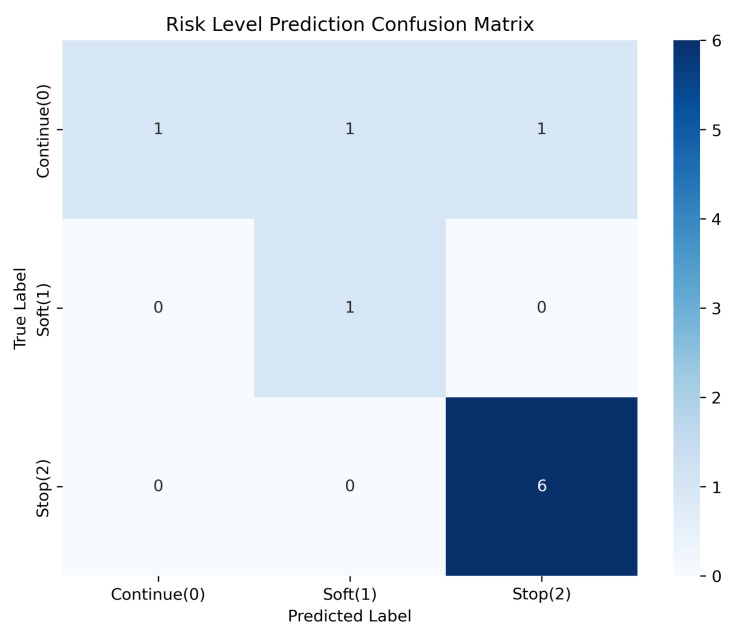
Performance on out-of-distribution vocabulary (N = 10).

We tested 10 samples containing terms not present in the training set (e.g., blooding, sleep, crying, scared, playing game). The performance is visualized in Figure 11, where the accuracy was 80.0% (8/10).

- The two errors were conservative (Continue classified as Soft or Soft classified as Stop).
- High-risk terms such as blooding and scared were mapped to the “Stop” action in these instances.

## 5. Semantic–Physical Fusion Interactive Control

The final stage maps the discrete semantic decisions (Action Tokens) from the LLM to continuous robot motion parameters. To ensure safety, the system modulates the reference trajectory parameters: Amplitude (*A*) and Period (*T*). The reference trajectory for the rehabilitation task is a sinusoidal motion:

(7) $$\theta_{\mathrm{ref}}\left(t\right)=A\cdot \sin\left(\frac{2\pi }{T}t\right)+\theta_{\mathrm{bias}}$$

where $\theta_{\mathrm{bias}}$ is the initial joint offset. The control framework adjusts *A* and *T* based on the decision output. Specifically, $\theta_{\mathrm{ref}}$ denotes the desired angular position of the human joint. To execute this motion, the control system maps $\theta_{\mathrm{ref}}$ to the Cartesian coordinates of the interaction points $s_{1}$ and $s_{2}$ (as defined in Figure 4) via the limb’s forward kinematics. The robot’s end-effectors are commanded to track these points while maintaining an orientation perpendicular to the limb segments. This kinematic mapping and the associated SE(3) transformations are computed in real-time using the Pinocchio library, ensuring precise coordination between the dual-arm system and the target limb configuration.

### 5.1. Action Space Definition

The LLM processes semantic information to generate control commands. We defined three discrete action modes for this experiment, although the framework can support larger action spaces.

- **Action 0 (Continue Training):** The robot executes the standard rehabilitation protocol.
  (8) $$A\to A_{\mathrm{default}},\;T\to T_{\mathrm{default}}$$
  where $A_{\mathrm{default}}$ and $T_{\mathrm{default}}$ are the standard amplitude and period parameters preset for the rehabilitation session. The robot maintains a standard rhythm (e.g., T=3 s) and full range of motion.
- **Action 1 (Soft Mitigation):** Triggered when the LLM determines that the situation is not dangerous but the user requires lower training intensity based on comprehensive semantic understanding. The objective is to dampen the interaction energy without stopping the training.
  (9) $$A^{\prime }=\gamma_{A}\cdot A_{\mathrm{default}}\;\left(0<\gamma_{A}<1\right)$$
  (10) $$T^{\prime }=\gamma_{T}\cdot T_{\mathrm{default}}\;\left(\gamma_{T}>1\right)$$
  In our experiments, we set $\gamma_{A}=0.5$ and $\gamma_{T}=1.6$. This results in a **smaller and slower** motion profile, allowing the user to recover control and reducing the risk of resonance with tremors.
- **Action 2 (Emergency Stop):** Triggered when the LLM determines that the user has an emergency risk requiring immediate cessation. The system immediately prioritizes safety by freezing the motion at the current position:
  (11) $$A\to 0,\;\theta_{\mathrm{bias}}\to \theta_{\mathrm{current}}$$
  By resetting the bias to the current angle ($\theta_{\mathrm{current}}$) while zeroing the amplitude, the trajectory is effectively flattened at the exact moment of the event, preventing the robot from dragging the user back to the center or causing secondary injury.

### 5.2. Experimental Setup

We conducted experiments involving a healthy subject simulating various rehabilitation scenarios. The hardware setup included:

- **Integrated Robot Platform:** A dual-arm upper limb rehabilitation robot integrated with 6-axis Force/Torque sensors and a built-in RGB-D camera for capturing multi-modal user states (Force, Facial, Posture).
- **Computational Unit (Laptop):** A laptop serving as the central processing station. It executes the visual recognition algorithms and hosts the fine-tuned LLM to perform real-time semantic reasoning and decision generation.
- **Seat Sensor:** A binary pressure switch put on the seat, providing definitive “On-Seat” or “Off-Seat” signals.

We designed four experimental scenarios (Table 3) to evaluate the system’s performance under conflicting or ambiguous conditions. These cases test how the LLM integrates diverse inputs—human posture, internal limb torque $\tau_{\mathrm{limb}\text{-}\mathrm{internal}}$, facial expression, and seat status—to select the appropriate safety strategy.

### 5.3. Results and Analysis

We analyzed the system’s decision making and control performance across the four designed scenarios. To verify repeatability, each experimental case was conducted with three trials. The system exhibited consistent behavior across all repetitions, correctly identifying safety levels and executing corresponding control actions in every trial. For clarity and conciseness, the results presented in the following figures correspond to a representative trial from each case. The results demonstrate how the fine-tuned LLM effectively maps multi-modal semantic tokens to appropriate safety protocols.

#### 5.3.1. Adaptive Control and Ambiguity Resolution (Cases A and B)

**Figure 12:**
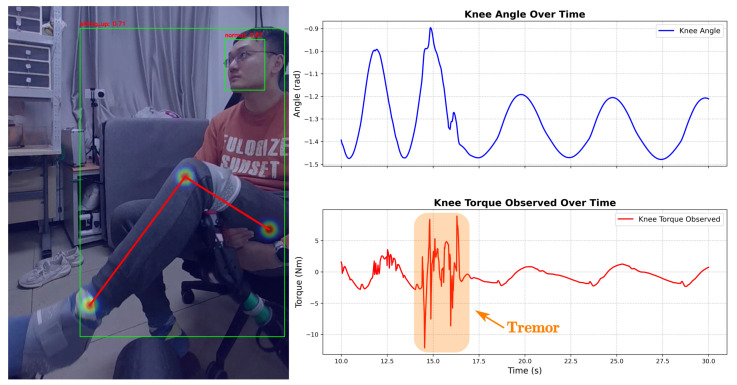
Adaptive compliance response to joint torque tremor (Case A).

Figure 12 details the system’s response to physiological disturbances (**Case A**). The force sensor detected high torque fluctuations (**Tremor**), while other sensors remained stable. The system interpreted this as muscle spasms. Consequently, **Action 1 (Soft Mitigation)** activated, reducing trajectory amplitude to accommodate the involuntary movements.

**Figure 13:**
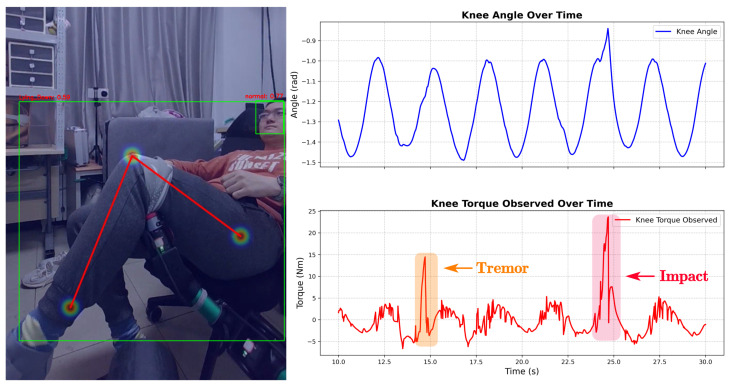
Accurate state determination via multi-modal semantic fusion (Case B).

In a traditional rule-based system, a “Lying” signal combined with a torque spike would typically trigger a fall alarm. However, as illustrated in Figure 13, the LLM output **Action 0 (Continue)**. This result highlights an emergent “black-box” behavior; the model appears to have implicitly correlated the torque transient with the benign postural shift (leaning back). Instead of reacting to the raw torque spike as a spasm, it filtered the disturbance as a mechanical side-effect of the voluntary movement, prioritizing the context (On Seat) over the instantaneous sensor fluctuation.

#### 5.3.2. Critical Safety Interventions (Cases C and D)

**Figure 14:**
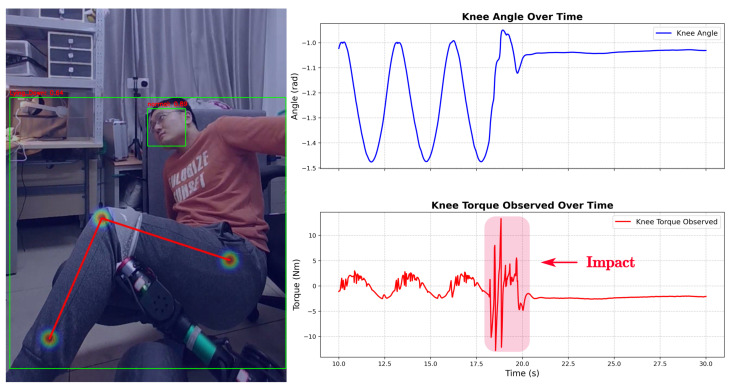
Immediate emergency stop triggered by a confirmed fall event (Case C).

**Case C** (Figure 14) presents a confirmed hazard. Unlike Case B, the visual “Lying” token coincided with a Seat: Off signal. This cross-validation confirmed a **Fall Event**. The model identified the high-risk pattern and executed **Action 2 (Emergency Stop)**, instantly latching the position to prevent secondary injury.

**Figure 15:**
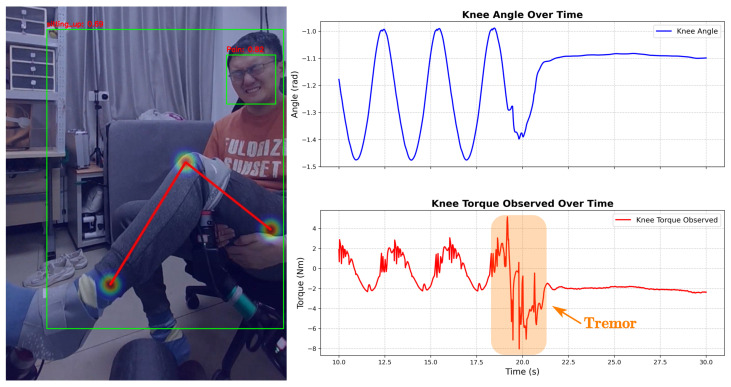
Safety intervention triggered by pain detection during tremor of joint torque (Case D).

**Case D** (Figure 15) tests the impact of **facial semantics** on safety logic. The physical signals (Tremor + On Seat) matched Case A exactly. However, the visual module detected a **Pain** expression. Although the user remained seated, the system recognized that tremor accompanied by pain indicates acute distress rather than simple fatigue. It escalated the response from ’Soft’ to **Action 2 (Emergency Stop)**, overriding the standard adaptive protocol.

### 5.4. Discussion: Contextual Reasoning and Safety Prioritization

The results indicate that the LLM-based control loop enables dynamic responses beyond rigid thresholds. We analyze this performance through two key mechanisms:

**Resolving Contextual Ambiguity (Case B vs. C):** Standard binary detectors often flag any “Lying” posture as a fall. The contrast between Case B and Case C demonstrates how the system differentiates context. By validating the visual input against the seat sensor, the model separates a dangerous fall (Lying + Off Seat) from a benign rest break (Lying + On Seat). This significantly reduces false alarms without compromising sensitivity to actual hazards. Crucially, this continuous pose monitoring supports flexible, pose-agnostic rehabilitation (e.g., bed-side vs. seated) suitable for unstructured home environments, rather than enforcing the rigid, static setups typical of clinical protocols.

**Prioritizing Human States over Physical Signals (Case A vs. D):** Comparing Case A and Case D illustrates the hierarchy of inputs. From a kinematic perspective, both cases present identical torque disturbances (Tremor) while the user is seated. A conventional admittance controller would likely react identically to both—simply softening the stiffness. However, the detection of “Pain” in Case D forces an Emergency Stop. The system prioritizes the semantic “Pain” signal over the kinematic “Tremor” signal, aligning the control strategy with clinical safety requirements rather than just signal processing logic.

In conclusion, the fusion strategy maps sensor data to semantic tokens, allowing the safety protocols to adapt to the broader context of the user’s state.

## 6. Conclusions and Future Work

In this paper, we presented a **Semantic–Physical Sensor Fusion** framework for dual-arm rehabilitation robots. The core novelty lies in bridging the gap between high-level intent understanding and low-level real-time control by unifying a dynamics observer with a Large Language Model (LLM).

Our experiments demonstrated that the “Seed-Augment-Train” pipeline is an effective strategy for adapting general-purpose LLMs to niche rehabilitation tasks. The fine-tuned model achieved 98.5% accuracy in intent detection and showed capability in handling unseen expressions. Crucially, the dynamics observer functioned as a reliable “virtual sensor,” enabling the system to detect torque anomalies even when visual data was occluded. On the execution side, the system successfully shifted from passive impedance adjustment to active **trajectory modulation**, triggering appropriate responses—from soft mitigation to emergency stops—with a control loop latency of approximately **0.223 s** on laptop.

Despite these promising results, several limitations remain to be addressed in future work:

1. **Dynamics Calibration:** While the current observer effectively detects relative anomalies (e.g., collisions), the absolute accuracy of torque estimation needs improvement through more rigorous parameter identification.
2. **Continuous Modulation:** We aim to move beyond the current discrete action space (Action 0/1/2) to continuous modulation coefficients. This would allow the LLM to generate smoother, non-binary assistance strategies based on the context.
3. **Hierarchical Architecture:** To balance reasoning depth with reaction speed, we plan to investigate a “Fast-Slow” architecture: employing Vision-Language-Action (VLA) models for complex, low-frequency reasoning, while retaining a lightweight Edge-LLM for high-frequency safety reflexes.

## Figures and Tables

**Table 1:** Hyperparameter settings for LoRA fine-tuning.

Parameter	Value
Base Model	Qwen3-1.7B
LoRA Rank (*r*)	8
LoRA Alpha (α)	32
Target Modules	q_proj,k_proj,v_proj,o_proj,gate,up,down
Dropout	0.1
Learning Rate	$3\times 10^{-4}$
Batch Size	16

**Table 2:** Classification performance report (N = 2000).

Class	Precision	Recall	F1-Score	Support
0 (Continue)	0.93	0.70	0.80	40
1 (Soft)	0.97	0.99	0.98	831
2 (Stop)	1.00	0.99	0.99	1129

**Table 3:** Experimental case design and LLM decisions.

Case	HPE	FER	Seat	Torque Disturbance	Action
A	Sitting	Normal	On	Yes (Tremor)	1 (Soft)
B	Lying	Normal	On	Yes (Impact)	0 (Continue)
C	Lying	Normal	Off	Yes (Impact)	2 (Stop)
D	Sitting	Pain	On	Yes (Tremor)	2 (Stop)

## Data Availability

The data presented in this study are available in this article.
